# Nuclear cardiology practice and associated radiation doses in Europe: results of the IAEA Nuclear Cardiology Protocols Study (INCAPS) for the 27 European countries

**DOI:** 10.1007/s00259-015-3270-8

**Published:** 2015-12-19

**Authors:** Oliver Lindner, Thomas N. B. Pascual, Mathew Mercuri, Wanda Acampa, Wolfgang Burchert, Albert Flotats, Philipp A. Kaufmann, Anastasia Kitsiou, Juhani Knuuti, S. Richard Underwood, João V. Vitola, John J. Mahmarian, Ganesan Karthikeyan, Nathan Better, Madan M. Rehani, Ravi Kashyap, Maurizio Dondi, Diana Paez, Andrew J. Einstein, for the INCAPS Investigators Group

**Affiliations:** Institute of Radiology, Nuclear Medicine and Molecular Imaging, Heart and Diabetes Center North Rhine-Westphalia Bochum, University Hospital of the Ruhr University, Georgstr. 11, 32545 Bad Oeynhausen, Germany; Section of Nuclear Medicine and Diagnostic Imaging, Division of Human Health, International Atomic Energy Agency, Vienna, Austria; Division of Cardiology, Department of Medicine, Columbia University Medical Center and New York-Presbyterian Hospital, New York, NY USA; Institute of Biostructures and Bioimaging, National Council of Research, Naples, Italy; Nuclear Medicine Department, Hospital de la Santa Creu i Sant Pau, Universitat Autónoma de Barcelona, Barcelona, Spain; Department of Nuclear Medicine and Cardiac Imaging, University Hospital Zurich, Zurich, Switzerland; Department of Cardiology, Sismanoglio Hospital, Athens, Greece; Turku PET Centre, University of Turku, and Turku University Hospital, Turku, Finland; National Heart and Lung Institute, Imperial College London, London, UK; Department of Nuclear Medicine, Royal Brompton and Harefield Hospitals, London, UK; Quanta Diagnóstico & Terapia, Curitiba, Brazil; Department of Cardiology, Houston Methodist DeBakey Heart and Vascular Center, Houston, TX USA; Department of Cardiology, All India Institute of Medical Sciences, New Delhi, India; Department of Nuclear Medicine, Royal Melbourne Hospital and University of Melbourne, Melbourne, Australia; Radiation Protection of Patients Unit, International Atomic Energy Agency, Vienna, Austria; Department of Radiology, Massachusetts General Hospital and Harvard Medical School, Boston, MA USA; Department of Radiology, Columbia University Medical Center and New York-Presbyterian Hospital, New York, NY USA

**Keywords:** Nuclear cardiology, Myocardial perfusion scintigraphy, SPECT, PET, Radiation dose, Best practices, Quality of care, Europe

## Abstract

**Purpose:**

Nuclear cardiology is widely used to diagnose coronary artery disease and to guide patient management, but data on current practices, radiation dose-related best practices, and radiation doses are scarce. To address these issues, the IAEA conducted a worldwide study of nuclear cardiology practice. We present the European subanalysis.

**Methods:**

In March 2013, the IAEA invited laboratories across the world to document all SPECT and PET studies performed in one week. The data included age, gender, weight, radiopharmaceuticals, injected activities, camera type, positioning, hardware and software. Radiation effective dose was calculated for each patient. A quality score was defined for each laboratory as the number followed of eight predefined best practices with a bearing on radiation exposure (range of quality score 0 – 8). The participating European countries were assigned to regions (North, East, South, and West). Comparisons were performed between the four European regions and between Europe and the rest-of-the-world (RoW).

**Results:**

Data on 2,381 European patients undergoing nuclear cardiology procedures in 102 laboratories in 27 countries were collected. A cardiac SPECT study was performed in 97.9 % of the patients, and a PET study in 2.1 %. The average effective dose of SPECT was 8.0 ± 3.4 mSv (RoW 11.4 ± 4.3 mSv; *P* < 0.001) and of PET was 2.6 ± 1.5 mSv (RoW 3.8 ± 2.5 mSv; *P* < 0.001). The mean effective doses of SPECT and PET differed between European regions (*P* < 0.001 and *P* = 0.002, respectively). The mean quality score was 6.2 ± 1.2, which was higher than the RoW score (5.0 ± 1.1; *P* < 0.001). Adherence to best practices did not differ significantly among the European regions (range 6 to 6.4; *P* = 0.73). Of the best practices, stress-only imaging and weight-adjusted dosing were the least commonly used.

**Conclusion:**

In Europe, the mean effective dose from nuclear cardiology is lower and the average quality score is higher than in the RoW. There is regional variation in effective dose in relation to the best practice quality score. A possible reason for the differences between Europe and the RoW could be the safety culture fostered by actions under the Euratom directives and the implementation of diagnostic reference levels. Stress-only imaging and weight-adjusted activity might be targets for optimization of European nuclear cardiology practice.

## Introduction

Nuclear cardiology is widely used to image myocardial perfusion and viability as well as left ventricular function noninvasively using SPECT or PET. The classical imaging procedure to diagnose coronary artery disease consists of stress imaging after radiopharmaceutical injection during dynamic exercise or pharmacological stress with adenosine, dipyridamole, regadenoson or dobutamine, and rest imaging. The radiopharmaceuticals used for SPECT are most commonly the ^99m^Tc-labelled compounds sestamibi or tetrofosmin and less commonly ^201^Tl-labelled compounds. The PET radiopharmaceuticals ^13^N-ammonia, ^82^Rb and ^15^O-water are used for PET perfusion imaging, and ^18^F FDG for viability imaging.

The information provided by nuclear cardiology can effectively diagnose coronary artery disease [1], stratify risk [2, 3], and guide patient management [4, 5], but it also exposes patients to the assumed risks of radiation exposure [6–8]. As imaging can be performed with several protocols, radiopharmaceuticals and additional techniques (e.g. attenuation correction), a variety of strategies and best practice procedures exist to obtain diagnostic quality images while minimizing exposure of the individual patient to radiation [9–11].

Information on current nuclear cardiology practice and radiation doses is scarce and mostly confined to single countries [11–13]. A worldwide study was therefore conducted during March and April 2013 to evaluate nuclear cardiology practice, and identify practices related to radiation dose and hence potential areas for improvement [14]. We present here the European data from the survey and a comparison with data from the rest-of-the-world (RoW).

## Materials and methods

### Study design and survey

This study used data collected as part of an IAEA cross-sectional study of nuclear cardiology laboratories around the world. Participating laboratories provided information about their nuclear cardiology practice from consecutive patients over one week between 18 March and 22 April 2013. Radiopharmaceuticals and administered activities in each laboratory were selected based on standard practice in that laboratory. During this period there was no ^99m^Tc generator shortage that may have impacted standard laboratory practice. A detailed description of the study design and data collection is published elsewhere [14].

A local investigator at each site provided data on laboratory and patient demographics and clinical characteristics for each patient undergoing a SPECT or PET nuclear cardiology procedure completed during the week. Procedures using planar techniques, e.g. multigated acquisition scans, were not included. The collected data included patient age, gender, weight, radiopharmaceuticals, injected activities, camera type, patient positioning, attenuation correction and image processing. Data omissions and errors were clarified individually with the laboratories.

The Columbia University Institutional Review Board approved the study, and deemed it exempt from the requirements of US federal regulations for the protection of human subjects (45 CFR 46) because no individually identifiable health information was collected.

### Radiation dose estimation

The primary outcome measure was patient effective dose, which was calculated from the radiopharmaceutical(s) administered and their activities, using the methodology outlined by the International Commission on Radiological Protection (ICRP), with the administered activity of each isotope multiplied by a conversion coefficient found in ICRP Publication 120 [15]. The only exception was ^82^Rb, for which the dose conversion coefficient of Senthamizhchelvan et al. [16], which is derived from more human data, was used to calculate the effective dose. We also evaluated the achievement in each laboratory of a median effective dose of ≤9 mSv, a target established in recommendations of the American Society of Nuclear Cardiology [17]. Radiation doses from imaging with attenuation correction, when performed, were not collected, since attenuation correction is generally associated with doses far less than those used in SPECT or PET imaging [18].

### Best practice quality score

Eight laboratory-level best practices pertaining to radiation dose according to current guidelines were determined a priori by an expert panel (Table 1) [10, 19, 20]. Each laboratory’s adherence to each practice was determined and a quality score for each laboratory was defined as the number of best practices out of the total of eight adhered to by that laboratory. A quality score of ≥6 was prespecified as a desirable level.

**Table 1:** Definition of the eight best practices

Item no.	Best practice	Definition	Basis for recommendation
1	Avoid ^201^Tl stress	No ^201^Tl studies performed in patients ≤70 years of age	SPECT imaging with ^201^Tl is associated with a considerably higher radiation dose to patients than ^99m^Tc [18]. This practice excludes ^201^Tl viability studies and stress-redistribution-reinjection stress-and-viability studies
2	Avoid dual isotope imaging	No dual isotope (rest ^201^Tl and stress ^99m^Tc) studies performed in patients ≤70 years of age	Dual isotope imaging is associated with the highest radiation dose of any protocol [18]
3	Avoid administration of too much ^99m^Tc	No study performed with ^99m^Tc activities >1,332 MBq (36 mCi), and mean total effective dose <15 mSv for all studies with two ^99m^Tc injections	1,332 MBq is the highest recommended activity in guidelines [21], and 15 mSv is a very high radiation dose for a ^99m^Tc study
4	Avoid administration of too much ^201^Tl	For each study with ^201^Tl, less than 129.5 MBq administered during stress	The expert committee maintained that 129.5 MBq should be the upper threshold for ^201^Tl activity
5	Perform stress-only imaging	At least one stress-only study performed, with rest imaging omitted, or only PET-based stress tests performed	If stress images are completely normal, subsequent rest imaging can be omitted
6	Use camera-based dose-reduction strategies	At least one study performed using at least one of the following: (1) attenuation correction (CT or transmission source), (2) imaging patients in multiple positions, e.g. both supine and prone, (3) high-technology software (e.g. resolution recovery and noise reduction), and (4) high-technology hardware (e.g. PET or a solid-state CZT SPECT camera)	Each of these approaches reduces the administered activity needed and facilitates performance of stress-only imaging
7	Use weight-based dosing for ^99m^Tc	Positive correlation between patient weight and administered activity (MBq) for injections of ^99m^Tc	Tailoring the administered activity to the patient weight offers an opportunity to reduce radiation dose
8	Avoid inappropriate dosing that can lead to “shine-through” artefact	No SPECT studies performed with ^99m^Tc rest and stress injections on the same day, in which the activity of the second injection was less than three times that of the first injection	Shine-through occurs in 1-day ^99m^Tc studies when residual radioactivity from the first injection interferes with the images following the second injection. To avoid shine-through, guidelines recommend that the activity of the second injection should be three to four times higher than that of the first injection. A second injection with an activity less than three times the activity of the first injection can lead to shine-through

A committee of international experts convened at the IAEA, including physicians and medical physicists, developed these criteria to be applied to nuclear cardiology laboratories. Each best practice, and thus also the quality score constituting the number of best practices adhered to, is defined for an individual laboratory, not for an individual patient. Adapted from Einstein et al. [14]

### Statistical methods

Mean (± standard deviation) and medians (interquartile range, IQR) were used to describe continuous variables, and were compared using analysis of variance and the Kruskal-Wallis test, respectively. The chi-squared test was used to compare categorical variables. The participating European countries were assigned to regions (North, East, South and West) according to the UN geoscheme (Table 2). Comparisons were performed among the four regions and between Europe and the RoW.

**Table 2:** Participating European countries and number of laboratories according to the UN geoscheme

East		North		South		West	
Country	No. of laboratories	Country	No. of laboratories	Country	No. of laboratories	Country	No. of laboratories
Czech Republic	3	Denmark	1	Bosnia and Herzegovina	1	Austria	5
Hungary	4	Estonia	1	Croatia	2	Belgium	2
Poland	6	Finland	1	Italy	25	France	1
Romania	1	Latvia	2	FYROM^a^	2	Germany	3
Slovakia	1	Lithuania	1	Portugal	4	Luxembourg	1
		Sweden	10	Serbia and Montenegro	2	Netherlands	1
		United Kingdom	15	Slovenia	2	Switzerland	2
				Spain	3		
Total	15		31		41		15

^a^Former Yugoslav Republic of Macedonia

The association between laboratory adherence to best practices and patient effective dose was evaluated using hierarchical linear regression models, accounting for clustering at the laboratory and country levels. Regression coefficients corresponded to the expected change in effective dose associated with adherence to a corresponding best practice. Patient effective dose was used as the dependent variable. The eight best practices were included as dichotomous (laboratory adherence, yes or no) independent variables and were treated as fixed factors. The intercept was defined as a random factor. Analyses were performed with and without adjustment for patient age, gender and weight. Correlations between model variables were assessed using Pearson’s *ϕ* coefficient. Statistical tests were considered significant with a two-tailed *P* value of <0.05. Analyses were performed using Stata/SE 13.1 (StataCorp, College Station, TX).

## Results

### Global Europe versus the RoW

Data were collected on 2,381 patients in 102 laboratories in 27 European countries with a mean of 23.3 ± 32.8 patients per laboratory (Table 2). Data from the RoW were from 5,530 patients, 206 laboratories and 38 countries with a mean of 26.8 ± 31.0 patients per laboratory. The list of the RoW countries is available elsewhere [14]. A cardiac SPECT study was performed in 2,330 (97.9 %) of the European patients (RoW 5,110, 92.4 %), and a cardiac PET study in 51 (2.1 %) of the European patients (RoW 420, 8.2 %).

The mean age of the European patients was 65.3 ± 11.1 years (RoW 63.7 ± 12.3 years, *P* < 0.001), and 40 % were women (RoW 41.7 %, *P* = 0.13). The mean effective dose from SPECT was 8.0 ± 3.4 mSv (RoW 11.4 ± 4.3 mSv, *P* < 0.001) and from PET was 2.6 ± 1.5 mSv (RoW 3.8 ± 2.5 mSv, *P* < 0.001). An effective dose of >9 mSv was received by 961 (41.2 %) of the European SPECT patients (RoW 3,874, 75.8 %, *P* < 0.001) and by none of all PET patients. Stress-only SPECT protocols were more frequent in Europe than in the RoW (19.7 % versus 10.6 %, *P* < 0.001). Demographics and effective doses are shown in Table 3.

**Table 3:** European demographics and effective doses versus the rest-of-the-world (RoW)

		Europe					Europe vs. RoW		
		East	North	South	West	*P* value	Europe	RoW	*P* value
Patients		261	583	1,019	518		2,381	5,530	
Women		131 (50.2 %)	246 (42.2 %)	357 (35.0 %)	215 (41.5 %)	<0.001	949 (39.9 %)	2,305 (41.7 %)	0.13
Age (years)									
Mean ± SD		63.0 ± 10.9	65.0 ± 12.2	65.6 ± 10.7	66.1 ± 10.7	<0.0001	65.3 ± 11.1	63.7 ± 12.3	<0.001
Median (interquartile range)		63 (56 – 72)	67 (57 – 74)	66 (58 – 73)	66 (59 – 74)	0.002	66 (58 – 74)	64 (55 – 73)	<0.001
Range		28 – 85	24 – 89	20 – 90	32 – 90		20 – 90	1 – 98	
SPECT	Patients	258	552	1,009	511		2,330	5,110	
	CZT	22	81	130	79	0.043	312	471	<0.001
	^201^Tl	8	24	3	31	<0.001	66	381	<0.001
	^99m^Tc	256	528	1,006	480	<0.001	2,270	4,881	<0.001
	Effective dose (mSv)								
	Mean ± SD	8.2 ± 4.1	6.9 ± 3.6	8.3 ± 2.9	8.7 ± 3.7	<0.001	8.0 ± 3.4	11.4 ± 4.3	<0.001
	Median	8.0	6.8	8.9	9.3	<0.001	8.1	11.5	<0.001
	Interquartile range	4.7 – 10.4	3.7 – 8.0	5.9 – 10.4	5.5 – 10.6		5.4 – 10.2	9.2 – 13.5	
	Patients with effective dose ≤9 mSv	152 (59.0 %)	445 (80.6 %)	536 (53.6 %)	236 (46.2 %)	<0.001	1,369 (58.8 %)	1,236 (24.2 %)	<0.001
PET	Patients	3	31	10	7		51	420	<0.001
	^18^F-FDG	3	0	5	0		8	32	
	^13^N-ammonia	0	0	5	7		12	33	
	^82^Rb	0	31	0	0		31	355	
	Effective dose (mSv)								
	Mean ± SD	7.3 ± 2.1	2.4 ± 0.2	2.7 ± 1.7	1.5 ± 0.7	<0.001	2.6 ± 1.5	3.8 ± 2.5	<0.001
	Median	8.6	2.5	2.5	1.2	0.002	2.5	3.6	<0.001
	Interquartile range	4.8 – 8.6	2.5 – 2.5	1.3 – 3.6	1.2 – 2.4		2.5 – 2.5	2.9 – 4.0	

Numbers for individual radioisotopes indicate number of patients who received at least one dose of the radioisotope

### Regional variation in Europe

European regions differed with respect to age and gender. In laboratories in the West region, patients were about 3 years older than in laboratories in the East region. The proportion of women undergoing testing varied from 35.0 % in the South region to 50.2 % in the East region. The mean numbers of patients in the observation week ranged from 17.4 to 34.5 per laboratory and did not differ significantly among the regions. Both mean and median effective doses from SPECT and from PET differed among the regions (*P* < 0.001 and *P* = 0.002, respectively). The effective dose from SPECT was lowest in the North region. The effective doses in the other regions were higher and similar to each other (*P* = 0.099; Table 3). Correspondingly, the proportion of studies with effective doses >9 mSv was higher in these regions (range 41.0 % to 53.8 %) and lowest in the North region (19.4 %, *P* < 0.001).

### SPECT protocols

Table 4 shows the numbers of SPECT stress-first and rest-first protocols. In Europe, significantly more stress-only protocols were performed than in the RoW (19.8 % vs. 8.2 %, *P* < 0.0001) although there was large variation among European centres (range 8.6 % to 33.4 %, *P* < 0.001). The South region had the lowest use of stress-only studies at 8.6 % with more common and similar use in the other regions (24.0 % to 33.4 %).

**Table 4:** SPECT protocols in Europe and the rest-of-the-world (RoW)

	East	North	South	West	Europe	RoW
No. of patients with stress study first	144	440	812	401	1,797	1,674
No. of patients with rest study first, then stress study	97	36	134	66	333	2,911
Total no. of patients with stress studies	241	476	946	467	2,130	4,585
No. of patients excluded because of undesirability of stress-only protocol^a^	20	107	73	51	251	945
No (%) of patients with stress-only protocol^b^	70 (29.0 %)	159 (33.4 %)	81 (8.6 %)	112 (24.0)	422 (19.8 %)	378 (8.2 %)

^a^Stress-only imaging would be clinically undesirable in some patients (e.g. viability study, rest-only study); therefore study was excluded from analysis of rate of stress-only studies

^b^ *P* < 0.001 within Europe, *P* < 0.0001 Europe vs. RoW

### Evaluation of quality scores

Quality scores are summarized in Tables 5 and 6. The mean European quality score was 6.2 ± 1.2, which was higher than that for the RoW (5.0 ± 1.1, *P* < 0.001). More European laboratories (70.6 %) adhered to six or more best practices than in the RoW (34.0 %, *P* < 0.001). Adherence to best practices did not vary among the European regions (range of mean quality score 6.0 to 6.4, *P* = 0.73).

**Table 5:** European quality scores versus the rest-of-the-world (RoW)

	Europe					Europe vs. RoW		
	East	North	South	West	*P* value	Europe	RoW	*P* value
No. of laboratories	15	31	41	15		102	206	
Score^a^								
1	0	0	0	0	n/a	0	0	n/a
2	0	0	0	0	n/a	0	2	n/a
3	0	0	0	0	n/a	0	15	n/a
4	1	4	2	3	0.007	10	46	0.007
5	3	5	10	2	0.004	20	73	0.004
6	6	6	11	5	0.89	28	54	0.817
7	4	7	14	2	<0.001	27	11	<0.001
8	1	9	4	3	<0.001	17	5	<0.001
≥6	11	22	29	10	0.99	72	70	<0.001
Mean ± SD	6.1 ± 1.0	6.4 ± 1.4	6.2 ± 1.1	6.0 ± 1.4	0.73	6.2 ± 1.2	5.0 ± 1.1	<0.001
Median	6	7	6	6	0.71	6	5	<0.001
Interquartile range	5 – 7	5 – 8	5 – 7	5 – 7		5 – 7	4 – 6	

^a^Number of best practices out of eight adhered to by a laboratory

**Table 6:** European best practices by region versus the rest-of-the-world (RoW)

	Europe					Europe vs. RoW		
	East	North	South	West	*P* value	Europe	RoW	*P* value
No. of laboratories	15	31	41	15		102	206	
Best practice								
Avoid ^201^Tl stress	15 (100 %)	30 (97 %)	40 (98 %)	12 (80 %)	0.078	97 (95 %)	185 (90 %)	0.13
Avoid dual isotope imaging	14 (93 %)	31 (100 %)	41 (100 %)	15 (100 %)	0.294	101 (99 %)	197 (96 %)	0.17
Avoid administration of too much ^99m^Tc	15 (100 %)	30 (97 %)	41 (100 %)	15 (100 %)	0.598	101 (99 %)	162 (79 %)	<0.001
Avoid administration of too much ^201^Tl	15 (100 %)	31 (100 %)	41 (100 %)	15 (100 %)	n/a	102 (100 %)	204 (99 %)	1
Perform stress-only imaging	4 (27 %)	17 (55 %)	18 (44 %)	8 (53 %)	0.308	47 (46 %)	46 (22 %)	<0.001
Use camera-based dose-reduction strategies	11 (73 %)	22 (71 %)	26 (63 %)	12 (80 %)	0.677	71 (70 %)	135 (66 %)	0.48
Use weight-based dosing for ^99m^Tc	5 (33 %)	17 (55 %)	21 (51 %)	5 (33 %)	0.365	48 (47 %)	40 (19 %)	<0.001
Avoid inappropriate dosing that can lead to “shine-through” artefact	12 (80 %)	20 (65 %)	26 (63 %)	8 (53 %)	0.514	66 (65 %)	70 (34 %)	<0.001

### Radiation dose and adherence to best practices

Imaging in a laboratory that adhered to each of the best practices was associated with a significantly lower effective dose with the exceptions of using weight-based dosing of ^99m^Tc (observed dose reduction not statistically significant), and avoiding inappropriate dosing that could lead to shine-through. This relationship was maintained after adjusting for patient age, gender and weight. Avoiding dual isotope use in patients <70 years of age was associated with the largest reduction in effective dose (9.51 mSv). The results of the hierarchical regression model adjusted for age, gender and weight are shown in Table 7. Pair-wise correlations between quality items were low. Pearson’s |*ϕ*| coefficient was less than 0.1 for most correlations, with modest correlations observed between sex and weight (0.3), and use of the best practices avoiding shine-through and stress-only imaging (0.4).

**Table 7:** Relationships between laboratory best practices and predicted patient effective dose from the final hierarchical regression model

Best practice	Reduction in predicted effective dose (mSv)			*P* value
	Mean	95 % confidence interval	Standard error	
Avoid ^201^Tl stress	4.55	2.34 – 6.75	1.13	<0.001
Avoid dual isotope imaging	9.51	4.89 – 14.1	2.36	<0.001
Avoid administration of too much ^99m^Tc	8.28	2.89 – 13.7	2.75	0.003
Avoid administration of too much ^201^Tl	Omitted because all laboratories followed this best practice			
Perform stress-only imaging	2.20	1.22 – 3.18	0.50	<0.001
Use camera-based dose-reduction strategies	1.13	0.15 – 2.11	0.50	0.023
Use weight-based dosing for ^99m^Tc	0.45	−0.51 – 1.42	0.49	0.356
Avoid shine-through	−1.04	−2.05 – −0.27	0.52	0.044
Age (years)	−0.003	−0.01 – 0.005	0.004	0.508
Female gender	0.44	0.25 – 0.62	0.09	<0.001
Weight (kg)	−0.04	−0.05 – −0.038	0.003	<0.001
Intercept (predicted effective dose)	28.7	21.1 – 36.2	3.84	<0.001

Regression model accounts for clustering within laboratory and country

## Discussion

With the growing incidence of cardiovascular disease [22], increasing use of nuclear cardiology is having an impact on patient exposure to radiation. This paper presents the data of the European subanalysis of the first worldwide study (INCAPS) on nuclear cardiology imaging protocols, associated effective doses and quality scores focusing on radiation exposure. Within the European Union, mandatory Council directives for medical exposure have been established since 1997 and implemented in the national laws of member states. The earlier directive 97/43/Euratom from June 1997 was replaced in December 2013 by the new Council directive 2013/59/Euratom which further delineates radiation protection standards for the European Union. Member states are committed to promoting the establishment and use of diagnostic reference levels (DRL) for radiodiagnostic examinations that creates a culture of radiation protection [23–25].

### Effective doses

The effective doses in Europe (mean 8.0 mSv; median 8.1 mSv, IQR 5.4 – 10.2 mSv) were lower than in the RoW, which could partly be because of the European regulations. Guidelines recommend that the median effective dose should be ≤9 mSv, and this was the case for European laboratories but there was variation among regions with the North having the lowest effective dose and only 19 % of patients receiving an effective dose above 9 mSv. Interestingly, effective doses in all the other regions were found to be very similar. The variability in effective doses in Europe can partly be explained by the different patterns in the use of PET and SPECT. Because of the short half-life of PET radiopharmaceuticals, the PET effective doses were lower than those used in SPECT, both in Europe and in the RoW. Given that PET is more sensitive for the detection of coronary artery disease [26], our data support efforts to increase access to and the use of cardiac PET. Patient effective doses must also be considered in the light of risk from cardiac disease, appropriateness of imaging and patient age. A recent analysis addressing these issues demonstrated that the long-term risk of patient exposure to radiation from noninvasive cardiac imaging is low and that the appropriate use of imaging the life-time risk of imaging procedures for fatal events is small compared with the risk of fatal cardiac events by coronary artery disease [27].

### Evaluation of quality scores and best practices

The worldwide INCAPS analysis found lower effective doses in patients who underwent cardiac SPECT and PET procedures in laboratories with better adherence to best practice [14]. Accordingly, adherence to radiation dose-related best practices was significantly better in Europe (quality score 6.2) than in the RoW (quality score 5.0). Although not statistically significant, quality parameters in the North European region were better than in the other regions which may explain the lower effective dose in Europe (Table 5). At least 17 % of all European laboratories followed all best practices (RoW 2 %), so 83 % have the potential, and according to the ALARA principle also the responsibility, to further increase their use of best practices.

As observed in multivariable modelling in the worldwide INCAPS study [14], in this European analysis the greatest benefit in terms of reducing dose was achieved through avoiding dual isotope imaging, followed by avoiding administration of too much ^99m^Tc, avoiding ^201^Tl stress testing, using stress-only imaging, and using camera-based does-reduction strategies. Stress-only imaging has been shown to reduce exposure of the individual patient to radiation by about 75 % if a 1-day stress–rest protocol was initially planned, or by about 50 % if a 2-day stress–rest protocol was planned. It entails performing the stress study first and then omitting the rest study in patients with unequivocally normal stress images. The protocol has been validated in large clinical studies and has no diagnostic disadvantages over routine acquisition of both stress and rest images [28]. Of the individual best practices, stress-only imaging was among the practices with low adherence in Europe and in the RoW. The South region of Europe had the lowest proportion of stress-only studies (8.6 %). In the North region the proportion was 30 % (Table 4). The 19 % of rest-first studies in Europe shows that there is the potential to increase the number of stress-only studies.

Similar reasoning applies to weight-based dosing, which was also one of the quality practices with low adherence. A recent study in three Italian centres has shown the effectiveness of this practice and indicates that factors to reduce effective doses have been recognized and are being implemented [11].

German surveys covering the years 2005 to 2012 have shown a decrease in effective dose from nuclear cardiology procedures over time and also that implementation of best practices takes time [13]. The changes in effective dose in Germany have been largely a result of the reduced use of ^201^Tl, which is now of only minor importance in Europe. As Table 6 shows, there are only a few laboratories that still use ^201^Tl or dual isotope imaging.

### Limitations

This study had several limitations. First, the number of responding European laboratories was modest, contributions from some European countries were missing, and for some regions the distribution of laboratories may have been imperfectly reflected by the distribution of participating laboratories; e.g. in the South region, Italy is either over-represented or has many more laboratories than other countries. The true number of laboratories practicing nuclear cardiology in Europe is unknown and it is unclear if the data are representative of overall European practice since responders may be a motivated subset of laboratories with good practice. Nevertheless, a comparison with current national survey data reveals that the data are plausibly representative [13, 29]. One implication of the uncertain response rate is the need to establish a database of European laboratories performing nuclear medicine procedures. Indeed, such a project is currently under consideration under EANM auspices. Second, the study involved only a single week, and thus, is potentially not representative of each laboratory’s practice.

Third, one can imagine rare individual clinical scenarios where non-adherence to some of the best practices identified by our expert panel is justified. However, we believe that careful attention to the specified best practices by a laboratory is indicative of reduced exposure to radiation of patients in the laboratory’s care, notwithstanding such exceptions. Nevertheless, as the quality score was derived from the best practices without weighting, caution should be used in interpreting the impact of the score on predicted effective dose. That is, in the case of a quality score <8 an identical score may have different implications on effective dose depending on the particular best practices followed. Fourth, the study focused on optimizing radiation dose and not on the justification for or appropriateness of nuclear cardiac testing. Radiation exposure can also be minimized by avoiding inappropriate examinations [18]. Finally, as INCAPS did not assess image quality, the relationships between radiation best practices and image quality could not be evaluated.

### Conclusion

While nuclear cardiology procedures have numerous benefits, they expose patients to ionizing radiation. The mean effective dose in a patient undergoing cardiac SPECT or PET in Europe is lower, and the quality score higher, than in the RoW. One reason for the European position may be the safety culture fostered by the Euratom directives for medical exposure implemented in the national laws of member states, the first in 1997 and the second in 2013. The use of stress-only protocols and weight-adjusted dosing of radiopharmaceuticals are potential targets for optimizing effective doses in patients undergoing nuclear cardiology imaging in Europe.

## Appendix

### Members of the INCAPS Investigators Group

**Executive Committee:** A.J. Einstein (chair), T.N.B. Pascual (IAEA project lead), D. Paez (IAEA section head), M. Dondi (IAEA section head); (alphabetically) N. Better, S.E. Bouyoucef, G. Karthikeyan, R. Kashyap, V. Lele, V.P.C. Magboo, F. Mut, J.J. Mahmarian, J.B. Meeks, M. Mercuri, M.M. Rehani, J.V. Vitola.

**Regional Coordinators:** (alphabetically) E. Alexánderson (Latin America), A. Allam (Africa/Middle East), M.H. Al-Mallah (Middle East), N. Better (Oceania), S.E. Bouyoucef (Africa), H. Bom (East Asia), A. Flotats (Europe), S. Jerome (United States), P.A. Kaufmann (Europe), V. Lele (South Asia), O. Luxenburg (Israel), J. Mahmarian (North America), L.J. Shaw (North America), S.R. Underwood (United Kingdom), J. Vitola (Latin America).

**Members:** (alphabetical order, by region) *Africa:* W. Amouri, H. Essabbah, S.S. Gassama, K.B. Makhdomi, G.I.E. El Mustapha, N. El Ouchdi, N. Qaïs, N. Soni, W. Vangu. *Asia:* R.M. Abazid, B. Adams, V. Agarwal, M.A. Alfeeli, N. Alnafisi, L. Bernabe, G.G. Bural, T. Chaiwatanarat, J.M. Chandraguptha, G.J. Cheon, I. Cho, A.S. Dogan, M. Eftekhari, A. Frenkel, I. Garty, S. George, P. Geramifar, H. Golan, S. Habib, R. Hussain, H. Im, H-J. Jeon, T. Kalawat, W.J. Kang, F. Keng, A. Klaipetch, P.G. Kumar, J. Lee, W.W. Lee, I. Lim, C.M.M. Macaisa, G. Malhotra, B.R. Mittal, M.H. Mohammad, P. Mohan, I.D. Mulyanto, D. Nariman, U.N. Nayak, K. Niaz, G. Nikolov, J.M. Obaldo, E. Ozturk, J.M. Park, S. Park, C.D. Patel, H.K. Phuong, A.P. Quinon, T.R. Rajini, Y. Saengsuda, J. Santiago, H.B. Sayman, A.S. Shinto, V. Sivasubramaniyan, M.H. Son, P. Sudhakar, G.M.S. Syed, N. Tamaki, K. Thamnirat, T. Thientunyakit, S. Thongmak, D.N. Velasco, A. Verma, U. Vutrapongwatana, Y. Wang, K.S. Won, Z. Yao, T. Yingsa-nga, R. Yudistiro, K.T. Yue, N. Zafrir. *Europe:* S.C. Adrian, D. Agostini, S. Aguadé, G. Armitage, M. Backlund, M. Backman, M. Baker, M.T. Balducci, C. Bavelaar, M. Berovic, F. Bertagna, R. Beuchel, A. Biggi, G. Bisi, R. Bonini, A. Bradley, L. Brudin, I. Bruno, E. Busnardo, R. Casoni, A. Choudhri, C. Cittanti, R. Clauss, D.C. Costa, M. Costa, K. Dixon, M. Dziuk, N. Egelic, I. Eriksson, G. Fagioli, D.B. de Faria, L. Florimonte, A. Francini, M. French, E. Gallagher, I. Garai, O. Geatti, D. Genovesi, L. Gianolli, A. Gimelli, E. del Giudice, S. Halliwell, M.J. Hansson, C. Harrison, F. Homans, F. Horton, D. Jędrzejuk, J. Jogi, A. Johansen, H. Johansson, M. Kalnina, M. Kaminek, A. Kiss, M. Kobylecka, M. Kostkiewicz, J. Kropp, R. Kullenberg, T. Lahoutte, O. Lang, Y.H. Larsson, M. Lázár, L. Leccisotti, N. Leners, O. Lindner, R.W. Lipp, A. Maenhout, L. Maffioli, C. Marcassa, B. Martins, P. Marzullo, G. Medolago, C.G. Mendiguchía, S. Mirzaei, M. Mori, B. Nardi, S. Nazarenko, K. Nikoletic, R. Oleksa, T. Parviainen, J. Patrina, R. Peace, C. Pirich, H. Piwowarska-Bilska, S. Popa, V. Prakash, V. Pubul, L. Puklavec, S. Rac, M. Ratniece, S.A. Rogan, A. Romeo, M. Rossi, D. Ruiz, N. Sabharwal, B.G. Salobir, A.I. Santos, S. Saranovic, A. Sarkozi, R.P. Schneider, R. Sciagra, S. Scotti, Z. Servini, L.R. Setti, S-Å. Starck, D. Vajauskas, J. Veselý, A. Vieni, A. Vignati, I.M. Vito, K. Weiss, D. Wild, M. Zdraveska-Kochovska. *Latin America:* R.N. Agüro, N. Alvarado, C.M. Barral, M. Beretta, I. Berrocal, J.F. Batista Cuellar, T-M. Cabral Chang, L.O. Cabrera Rodríguez, J. Canessa, G. Castro Mora, A.C. Claudia, G.F. Clavelo, A.F. Cruz Júnior, F.F. Faccio, K.M. Fernández, J.R. Gomez Garibo, U. Gonzalez, P. González E., M.A. Guzzo, J. Jofre, M. Kapitán, G. Kempfer, J.L. Lopez, T. Massardo V., I. Medeiros Colaco, C.T. Mesquita, M. Montecinos, S. Neubauer, L.M. Pabon, A. Puente, L.M. Rochela Vazquez, J.A. Serna Macias, A.G. Silva Pino, F.Z. Tártari Huber, A.P. Tovar, L. Vargas, C. Wiefels. *North America:* A. Aljizeeri, R.J. Alvarez, D. Barger, W. Beardwood, J. Behrens, L. Brann, D. Brown, H. Carr, K. Churchwell, G.A. Comingore, J. Corbett, M. Costello, F. Cruz, T. Depinet, S. Dorbala, M. Earles, F.P. Esteves, E. Etherton, R.J. Fanning, Jr., J. Fornace, L. Franks, H. Gewirtz, K. Gulanchyn, C-L. Hannah, J. Hays, J. Hendrickson, J. Hester, K. Holmes, S. Jerome, A. Johnson, C. Jopek, H. Lewin, J. Lyons, C. Manley, J. Meden, S. Moore, W.H. Moore, V. Murthy, R. Nace, D. Neely, L. Nelson, O. Niedermaier, D. Rice, R. Rigs, K. Schiffer, E. Schockling, T. Schultz, T. Schumacker, B. Sheesley, A. Sheikh, B. Siegel, A.M. Slim, J. Smith, M. Szulc, N. Tanskersley, P. Tilkemeier, G.D. Valdez, R. Vrooman, D. Wawrowicz, D.E. Winchester. *Oceania:* A. Alcheikh, B. Allen, E. Atkins, J. Bevan, C. Bonomini, J. Christiansen, L. Clack, E. Craig, H. Dixson, I. Duncan, S. Fredericks, S. Gales, R. Hampson, T. Hanley, K. Hartcher, J. Hassall, B. Kelley, S. Kelly, T. Kidd, T. de Kort, G. Larcos, W. Macdonald, C. McGrath, E. Murdoch, S. O’Malley, M. O’Rourke, M. Pack, R. Pearce, R. Praehofer, S. Ramsay, L. Scarlett, K. Smidt, F. Souvannavong, K. Taubman, G. Taylor, K. Tse, S. Unger, J. Weale.
